# Behavioral flexibility is associated with changes in structure and function distributed across a frontal cortical network in macaques

**DOI:** 10.1371/journal.pbio.3000605

**Published:** 2020-05-26

**Authors:** Jérôme Sallet, MaryAnn P. Noonan, Adam Thomas, Jill X. O’Reilly, Jesper Anderson, Georgios K. Papageorgiou, Franz X. Neubert, Bashir Ahmed, Jackson Smith, Andrew H. Bell, Mark J. Buckley, Léa Roumazeilles, Steven Cuell, Mark E. Walton, Kristine Krug, Rogier B. Mars, Matthew F. S. Rushworth

**Affiliations:** 1 Wellcome Centre for Integrative Neuroimaging, Department of Experimental Psychology, University of Oxford, Oxford, United Kingdom; 2 Univ Lyon, Université Lyon 1, Inserm, Stem Cell and Brain Research Institute U1208, Bron, France; 3 Wellcome Centre for Integrative Neuroimaging, Centre for Functional MRI of the Brain (FMRIB), Nuffield Department of Clinical Neurosciences, University of Oxford, John Radcliffe Hospital, Oxford, United Kingdom; 4 National Institute of Mental Health, Magnuson Clinical Center, Bethesda, Maryland, United States of America; 5 McGovern Institute for Brain Research and Department of Brain and Cognitive Sciences, Massachusetts Institute of Technology, Cambridge, Massachusetts, United States of America; 6 Department of Physiology, Anatomy and Genetics, University of Oxford, Oxford, United Kingdom; 7 Otto-von-Guericke-Universität, Magdeburg, Germany; 8 Leibniz-Institut für Neurobiologie, Magdeburg, Germany; 9 Donders Institute for Brain, Cognition and Behaviour, Radboud University Nijmegen, Nijmegen, the Netherlands; University of Minnesota, UNITED STATES

## Abstract

One of the most influential accounts of central orbitofrontal cortex—that it mediates behavioral flexibility—has been challenged by the finding that discrimination reversal in macaques, the classic test of behavioral flexibility, is unaffected when lesions are made by excitotoxin injection rather than aspiration. This suggests that the critical brain circuit mediating behavioral flexibility in reversal tasks lies beyond the central orbitofrontal cortex. To determine its identity, a group of nine macaques were taught discrimination reversal learning tasks, and its impact on gray matter was measured. Magnetic resonance imaging scans were taken before and after learning and compared with scans from two control groups, each comprising 10 animals. One control group learned discrimination tasks that were similar but lacked any reversal component, and the other control group engaged in no learning. Gray matter changes were prominent in posterior orbitofrontal cortex/anterior insula but were also found in three other frontal cortical regions: lateral orbitofrontal cortex (orbital part of area 12 [12o]), cingulate cortex, and lateral prefrontal cortex. In a second analysis, neural activity in posterior orbitofrontal cortex/anterior insula was measured at rest, and its pattern of coupling with the other frontal cortical regions was assessed. Activity coupling increased significantly in the reversal learning group in comparison with controls. In a final set of experiments, we used similar structural imaging procedures and analyses to demonstrate that aspiration lesion of central orbitofrontal cortex, of the type known to affect discrimination learning, affected structure and activity in the same frontal cortical circuit. The results identify a distributed frontal cortical circuit associated with behavioral flexibility.

## Abstract 2

The classical idea that central orbitofrontal cortex (cOFC) mediates behavioral flexibility has been recently challenged. This study provides further such evidence, identifying a neural network associated with behavioral flexibility centered on lateral OFC/anterior insula and subcortical regions such as the amygdala.

## Introduction

One of the most influential accounts of orbitofrontal cortex (OFC) function suggests that it mediates behavioral flexibility in response to changes in the environment [1–4]. Typically, this has been assessed with discrimination reversal (DisRev) learning tasks in which animals learn one choice that leads to reward, whereas another does not. Usually the correct and incorrect choices are defined as the selection of one stimulus rather than another, but sometimes spatially defined choices are employed. Animals learn to make the reward-associated choice, but once they make it reliably, the reward assignments are switched so that the previously unrewarded choice becomes the only one followed by reward. Central OFC lesions centered on the orbital gyrus (areas 13 and 11) have long been thought to impair DisRev, and the activity of OFC neurons has been related to key DisRev events [1,4–8].

The consensus view of OFC function has recently been questioned by the finding in macaques that, although DisRev is impaired by aspiration lesions of central OFC, it is unimpaired if the lesions are made with excitotoxic injections sparing fibers of passage [3]. The implication is that behavioral flexibility in DisRev performance depends on a specific network of brain regions that are disconnected from one another by aspiration lesions of OFC (which may damage fibers of passage) but not by excitotoxic lesions. The identities of the components of this circuit are, however, unknown.

One key region of interest (ROI) is the cortex in the lateral OFC (lOFC). In the past, the term lOFC has sometimes been used to refer to any cortex lateral to the medial orbital sulcus, but here, we use it more specifically to refer just to the cortex in and lateral to the lateral orbital sulcus. This corresponds to the orbital part of area 12 (12o). This region is important for the linking of a choice to an outcome and using knowledge of such linkages to guide behavior. For example, activity here reflects the use of a win–stay/lose–shift behavioral strategy [9]. Win–stay/lose–shift strategies require specific outcome events to be linked to specific choices, which are then repeated or avoided in the future depending on how successful they have been. The corresponding region in the human brain [10] has been linked to the learning of specific choice–reward associations [11–13]. Decisions are no longer driven by knowledge of the causal relationships between choices and outcomes when lesions are made that include this region and adjacent ventrolateral prefrontal cortex in the rhesus macaque [14,15]. Here, we refer to the adjacent cortex lying between the lateral and medial orbital sulci as the central OFC (areas 11 and 13) and the cortex medial to the medial orbital sulcus as the medial OFC (mOFC)/ventromedial prefrontal cortex (vmPFC).

Another potentially important candidate region is the posterior OFC and adjacent anterior insula (AI); Rudebeck and colleagues [3] showed that small aspiration lesions nearby, placed across the posterior OFC, were sufficient to impair DisRev. However, one interpretation of the finding is that damage to white matter pathways in the vicinity, such as the uncinate fascicle, extreme capsule, and cingulum bundle [16,17], cause the DisRev impairment. Many prefrontal regions, including posterior OFC and AI, are interconnected by these pathways.

An additional reason for thinking that AI and posterior OFC might be important for DisRev is that OFC lesions in new-world monkeys and rats, even when made by excitotoxin injection, have been reported to cause DisRev impairments [4,8,18–21]. It is possible that the regions referred to as OFC in rodents and new-world monkeys have similarities with the posterior OFC and adjacent AI of old-world primates such as macaques [22,23]. Secondly, Wittmann and colleagues [68] have recently shown that activity in this region of the macaque brain reflects not just the outcome of the last choice made but also a longer-term history of reward. An animal learns to balance the weight of influence exerted by these signals when it becomes proficient at performing DisRev.

There are other regions within the network of areas thought to be important for reward-guided decision-making that have connections that might be compromised by the white matter damage after OFC aspiration lesion. These include the anterior part of the cingulate cortex, which is involved in tracking key features of the reward environment [24–28] and holding the value of alternative or counterfactual choices in order to guide changes in behavior [29–31].

To identify the wider network of brain regions involved in DisRev learning, we carried out a series of behavioral, lesion, and neuroimaging experiments in macaques and examined changes in gray matter and functional connectivity. Learning-related plasticity has been shown to occur at different timescales and at different levels (for review, see [32–36]). It has been studied in the context of perceptual learning [37], motor learning [38,39], and in more-cognitive domains [40,41]. The nature of the training has been shown to affect distinct circuits [42]. Although the development of imaging techniques provides a potentially noninvasive route for demonstrating changes at the circuit level, there is still debate about the biological nature of the changes. Several non–mutually exclusive hypotheses have been proposed, including axon sprouting, dendritic branching and synaptogenesis, changes in glial number and morphology, and angiogenesis [33]. Although it might be difficult to identify changes in dendritic spines with magnetic resonance imaging (MRI), it is likely that MRI is sensitive to angiogenesis and glial changes that occur at the same time. Sampiao-Baptista and colleagues [38,43] used MRI to demonstrate gray matter changes in rodents in a motor learning task that had previously been used to demonstrate synaptic changes [44]. Importantly, the task used in both of these studies made it possible to compare a motor learning condition with a control condition matched for motor activity level but lacking a learning element. MRI-measured gray matter changes and synaptic changes are found when learning occurs. MRI studies suggest that gray matter changes happen quickly [45,46], as do rodent studies of spine changes [47]. We, therefore, hypothesize that the specific recruitment of circuits to support reversal learning will be associated with increases in gray matter volumes that can be measured using deformation-based morphometry (DBM) [38,39,42,48–50] and by a strengthening of activity coupling between brain regions [48,49].

First, in experiment 1, we carried out a longitudinal experiment in which we sought brain regions where structural changes were associated with DisRev learning in a group of nine macaques. Structural MRI scans under anesthesia were taken before and after DisRev learning and were compared with matched scans from two groups of control animals taught to perform either a similar reward-guided discrimination task that lacked any reversal component (10 animals) or no task at all (10 animals). Next, to identify regions affected by transneuronal degeneration caused by central OFC aspiration lesions, we used similar techniques to determine whether changes occurred in the same neural circuit when nonfiber sparing lesions were made in central and mOFC in two macaques. Gray matter throughout the brain in these two animals was compared with gray matter throughout the brain in MRI scans from 28 control macaques. Finally, in experiments 3 and 4, we examined changes in functional activity within the areas identified in experiments 1 and 2. Equivalent analyses were performed in functional MRI (fMRI) scans taken before and after learning (experiment 3), as well as in lesion and control animals (experiment 4). We sought converging evidence for both brain regions affected by the aspiration lesion of OFC in experiment 2 and brain regions in which structure and activity were modulated by DisRev learning in experiments 1 and 3, reasoning that effects that replicated across the different studies would be the most reliable.

## Results

### Experiment 1

**Fig 1:**
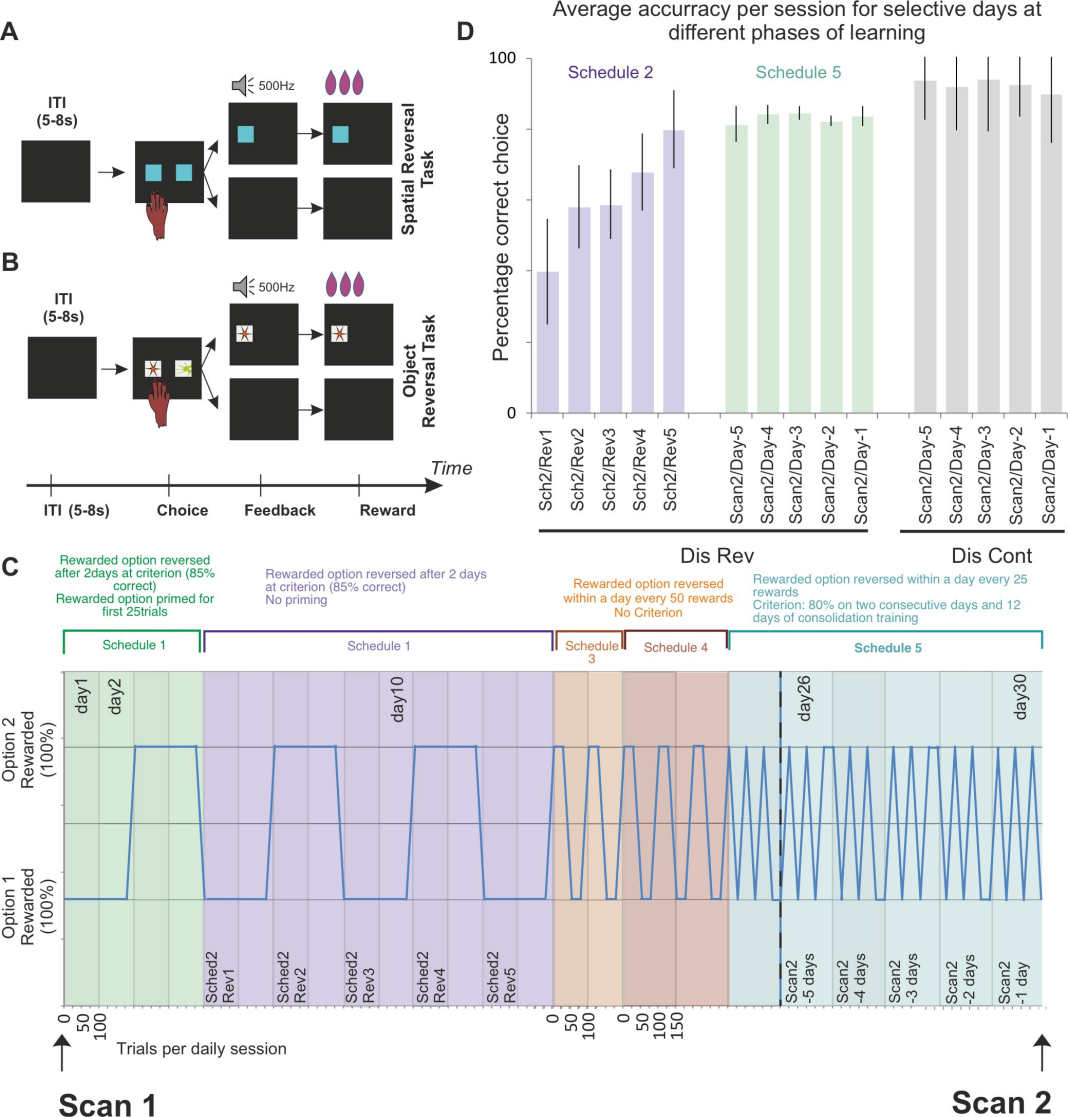
Trial structure in (A) spatial DisRev, (B) object DisRev, and (C) overview of DisRev experience between structural scan 1 and 2 in a longitudinal investigation of the effect of learning on brain structure and function. The schematic illustrates an ideal performer. The minimum number of trials needed by an ideal performer per daily session is illustrated on the abscissa, and reward properties of the two options are plotted on the ordinate. All reversal tasks were deterministic (in any given period, one option was always rewarded when chosen, and the other option was never rewarded when chosen). In the first testing schedules, animals completed 100 trials per day, and one of two simultaneously presented options was designated the rewarded target for the day. In schedule 1, the rewarded choice was primed for 25 trials before introducing, in addition, the unrewarded choice on the opposite side of the screen for an additional 75 trials. The choice–reward contingencies were reversed the day after each animal performed above 85% correct for two consecutive days. Schedule 2 was similar, but now the rewarded choice was not primed. Animals experienced five reversals under schedule 2. With perfect performance, an animal could, therefore, complete this second phase in 10 days. Schedules 3 and 4 introduced a choice–reward association reversal within a given day’s testing session; the reversal occurred once the animal had correctly chosen the target 50 times. The animals completed 2 days of 100 trials and 3 days of 150 trials on schedules 3 and 4, respectively. There was no performance criterion in these phases. As such, schedules 3 and 4 were not goals in themselves but stepping stones toward the more volatile reward environment of schedule 5. In the final schedule, the rewarded targets reversed after 25 correctly performed trials. In total, the animals had to perform 150 correct trials in a day. Their second scan took place once performance exceeded 80% correct for two consecutive days and after 12 subsequent days of consolidation training. (D) Average correct performance over session for the first five sessions they encountered a reversal (schedule 2 sessions; see Fig 1C) and for the last 5 days prior to the second scan (schedule 5 sessions). During schedule 2, monkeys performed at approximately 55% accuracy, but by schedule 5 reversal, they performed at 80% accuracy. Data are available on https://www.jeromesallet.org/data-ofc-reversal-learning and https://doi.org/10.5281/zenodo.3776631. Dis Cont, discrimination control; DisRev, discrimination reversal; ITI, intertrial interval; Rev, reversal; Sch, schedule; Sched, schedule.

First, we investigated the wider structural network of brain regions affected by DisRev learning. We sought brain regions in which structural (experiment 1) and activity (experiment 3) changes were associated with DisRev experience in a group of nine macaques. Four animals chose between stimuli with different identities (object DisRev), and five chose between different target locations (spatial DisRev). In both cases, the tasks were deterministic; at any given time, one choice was rewarded, and the other choice was not rewarded. We obtained structural MRI and fMRI measures at two time points before and after behavioral training (pre and post-learning) while animals were under general anesthesia (Fig 1). At scan 1, the animals had some experience with the experimental apparatus and had learned that pressing a target on either the left or right of a touchscreen was associated with a juice reward. By contrast, at scan 2, animals had substantial experience of reversal tasks and could perform five reversals per day, every time 25 correct choices had been made, at high levels of accuracy (≥80% correct on average throughout the entire session). Learning has previously been associated with structural and activity changes that can be measured with MRI, even when subjects are at rest [33]. Typically, such investigations have focused on sensorimotor learning and, consequently, on sensorimotor brain regions, but given the connectional changes observed in rodents during learning [40], there is no reason why the learning of a “cognitive set” for behavioral flexibility may not be associated with similar structural and activity changes in other brain regions.

A general linear model (GLM) was used to compare gray matter in the nine DisRev learning macaques at scan 1 and scan 2 (mean 0.71 years, 0.28 SD) with that in similarly spaced scans (mean 0.98 years, 0.76 SD) in 20 control macaques that lacked experience of DisRev (S1 Table). Half the control animals (*n* = 10) had no experience of formal training (no-discrimination control [NoDis Control]), whereas the other half (*n* = 10) had experience of discriminating visual stimuli but in the absence of any reversal requirement (discrimination control [Dis Control]). There was no significant difference in the number of days between scan 1 and scan 2 when DisRev learners and all controls were compared (t_27_ = 1.03, *p* = 0.312). Structural MRI data were submitted to a DBM analysis using the Oxford Centre for Functional Magnetic Resonance Imaging (FMRIB) Software Library (FSL) tools FNIRT and Randomise [51] (see Methods). The logic of the approach is that if a group of brain images can be warped to an identical image, then volumetric changes involved in that warping process give measures of the local differences in brain structure between individuals. Related analyses have previously been described [48,49]. In addition to our regressor of interest (behavioral condition: DisRev learners, NoDis Control, Dis Control) we also included control regressors indexing the age and sex of individual monkeys.

At each voxel in the brain, the dependent variable was the determinant of the Jacobian matrix from the nonlinear registration of each individual’s structural MRI to the group average brain. This is a scalar variable representing how much each voxel in an individual’s brain would need to be expanded or compressed to match the group average brain. To check the reliability of our findings, we sought regions in which effects were replicated in both hemispheres by testing for the conjoint probability of symmetrical effects in the two hemispheres with a *p* < 0.001 (under the null hypothesis, effects are expected to be randomly distributed across hemispheres [52]) and constituted by more than 15 contiguous voxels (see Methods). In examining the bilaterality of our effects, we adopt an approach suggested for MRI voxel-based gray matter analyses. It is based on the principle that taking into account the spatial extent, across adjacent MRI voxels, of any statistical effect is not necessarily appropriate for gray matter analyses [53], and so alternative tests of robustness have been advocated that involve examining whether effects are bilaterally symmetrical [49,54,55]. The premise rests on the assumption that if a statistical effect noted had a chance of occurrence of *p* = 0.05 in one brain area under the null hypothesis, then it has the chance of occurring in the same area in both hemispheres with the square of this probability (e.g., *p* = 0.05 × 0.05 = 0.0025). As explained above, we report here bilaterally symmetrical effects with a conjoint probability of *p* < 0.001.

**Fig 2:**
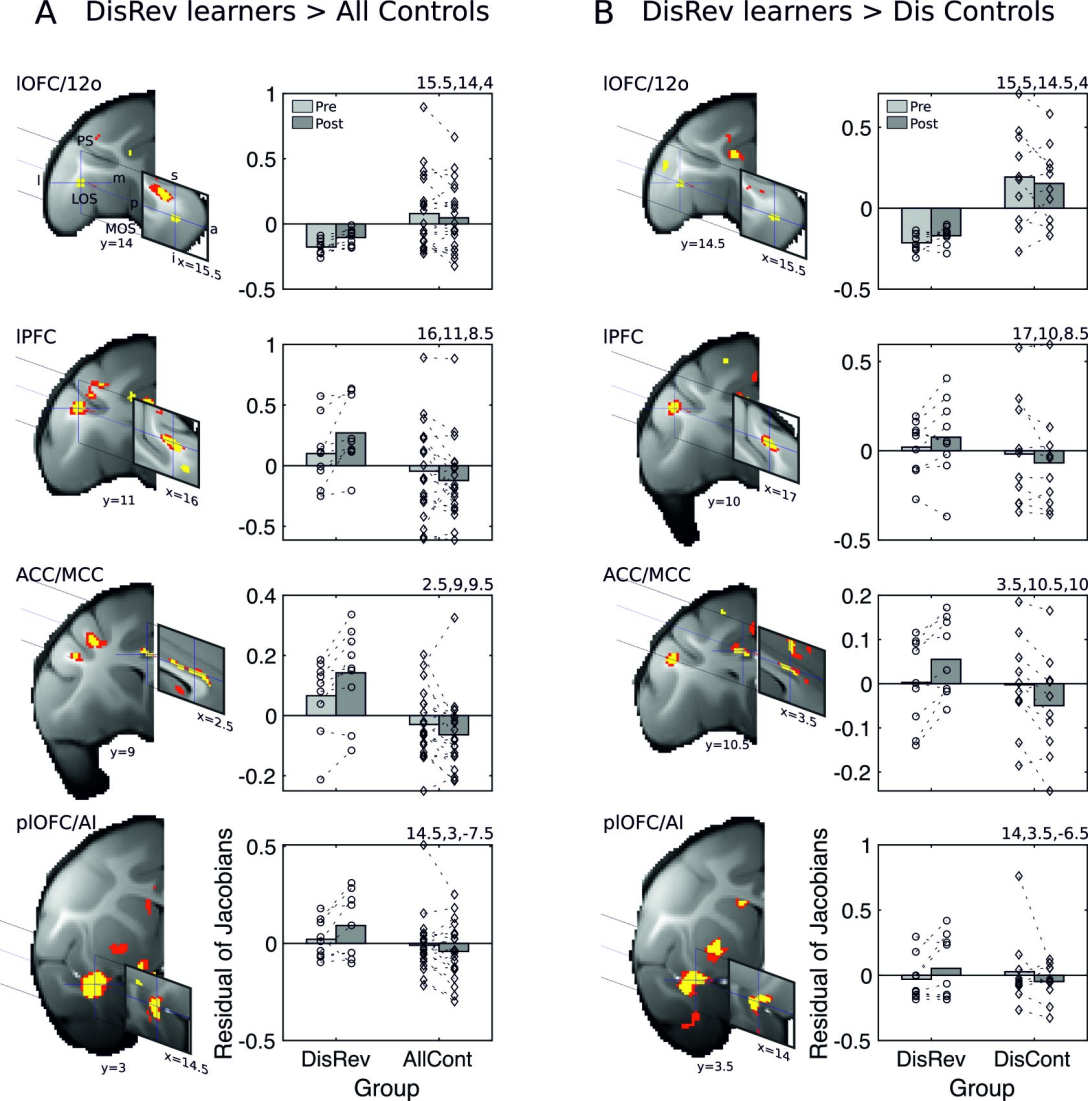
Learning DisRev was associated with distributed gray matter increments (A) relative to all control animals, including in the same set of brain regions as identified in experiment 2: lOFC/12o (cross hairs in top row), ventral bank of the PS in anterior lPFC (cross hairs in second row), ACC/MCC (cross hairs in third row), and plOFC/AI (cross hairs in bottom row). Panel B illustrates that excluding NoDis Controls from the analysis revealed the same set of brain regions with increases in gray matter in lOFC/12o, ventral bank of the PS, ACC/MCC, and plOFC/AI. Results of analysis testing for bilaterally symmetrical effects (*p* < 0.001) are shown in yellow and red (*p* < 0.005). Across analyses, gray matter shows relative increases after learning in the DisRev animals and relative decreases in the NoDis Controls or Dis Controls. Because in the initial stages of analysis, all scans from both before and after training are registered to a template derived from their group average, the baseline residual Jacobian values in each figure lie close to the mean. 12o, orbital part of area 12; a, anterior; ACC/MCC, anterior cingulate cortex/midcingulate cortex; AI, anterior insula; AllCont, all control; Dis Control, discrimination control; DisRev, discrimination reversal; I, inferior; l, lateral; lOFC, lateral OFC; lPFC, lateral prefrontal cortex; m, medial; LOS, lateral orbital sulcus; MOS, medial orbital sulcus; NoDis Control, no-discrimination control; OFC, orbitofrontal cortex; p, posterior; plOFC, posterior lOFC; plOFC/AI, plOFC extending into AI; PS, principal sulcus; s, superior. Data are available on https://www.jeromesallet.org/data-ofc-reversal-learning and https://doi.org/10.5281/zenodo.3776631.

Significant changes in gray matter in several regions were observed (Fig 2A, S3 and S2 Tables). We focus here on those regions of significant change that were also identified as important in the very different approach undertaken in experiment 2. Significant gray matter increases were associated with DisRev experience compared with all controls in four parts of the frontal lobes, including the anterior part of a region that we have previously referred to as lOFC [9–11] but is more unambiguously identified by the term 12o, posterior lOFC (plOFC) extending into AI (plOFC/AI), ventral bank of the principal sulcus in the lateral prefrontal cortex (lPFC), and in the anterior part of cingulate cortex (ACC) close to the region referred to as midcingulate cortex (MCC) by Procyk and colleagues [56]. We refer to it here as ACC/MCC. For illustration, we present the averaged residual Jacobian values extracted from bilateral ROIs placed at the center of gravity of the significant clusters.

Further post hoc tests examined whether effects reflected increases in gray matter in DisRev learners or decreases in controls. A session (two levels: scan 1 versus scan 2) × area (four levels: lOFC/12o, plOFC/AI, lPFC, ACC/MCC) × hemisphere (two levels: right and left) × structural scan (two levels) analysis found that gray matter across ROIs tended to increase in learners (main effect of session: F_1,8_ = 19.51, *p* = 0.002) while reducing in all controls (main effect of session: F_1,19_ = 5.26, *p* = 0.033).

To confirm that the effects were not dependent on nonspecific effects associated simply with learning to discriminate between visual stimuli, as opposed to the specific effect of reversing reward contingencies, we examined the contrast between the DisRev learners and Dis Control (and excluded the NoDis Control animals). Importantly, the effects are not driven by differences in performance. We did not observe significant differences in the performances of the DisRev and Dis Controls on the five sessions that preceded scan 2 (t_17_ = −1.0041, *p* = 0.3294). Next, we compared gray matter in the DisRev and Dis Control groups; significant gray matter increases were notably associated with DisRev experience bilaterally (*p* < 0.001) in the same regions (lOFC/12o, plOFC/AI, lPFC, ACC/MCC [Fig 2B; see S3 Table]). Again, we illustrate the averaged residual Jacobian values extracted from bilateral ROIs placed at the centers of gravity of the significant clusters. In summary, the changes seen in these four regions—lOFC/12o, plOFC/AI, lPFC, and ACC/MCC—are linked to the reversal learning process, which distinguishes the DisRev and Dis Control groups.

As a second test of the specificity of the effects reported in the four ROIs, we examined whether gray matter in these areas differed when the two control groups (Dis Control and NoDis Control) were compared. We conducted a five-way repeated measures ANOVA including factors of session (two levels: pre, post) × area (four levels: frontal ROIs) × hemisphere (two levels: left and right) × structural scan (two levels) × group (two levels: Dis Controls, NoDis Controls). This analysis failed to identify an interaction between session and group (F_1,18_ = 0.169, *p* = 0.686) or any other subsidiary interactions including session and group (all *p*’s > 0.292).

**Fig 3:**
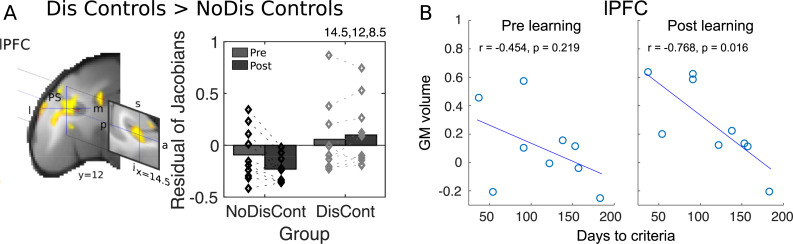
(A) lPFC GM volume is greater in control animals who experienced discrimination learning (Dis Cont) relative to control animals who had learning experience (NoDis Cont) between scan 1 and scan 2. (B) lPFC GM volume before (left panel) and after (right panel) DisRev learning training (DisRev learners) correlated with the number of training days the animals required to reach performance criterion. The negative relationship between lPFC GM volume and performance, although evident before learning, only becomes significant after extended experience of DisRevs. a, anterior; Dis Cont, discrimination control; DisRev, discrimination reversal; GM, gray matter; I, inferior; l, lateral; lPFC, lateral prefrontal cortex; m, medial; NoDis Cont, no-discrimination control; p, posterior; PS, principle sulcus; s, superior. Data are available on https://www.jeromesallet.org/data-ofc-reversal-learning and https://doi.org/10.5281/zenodo.3776631.

It is, of course, possible that gray matter changes may occur in relation to simple discrimination task performance in other brain areas outside or partially overlapping with the four frontal ROIs that were identified by the comparison of DisRev learners with controls. DisRev tasks particularly tax choice–reward learning mechanisms because they repeatedly require relearning of choice–reward associations, but the same learning mechanisms are thought to be set in play by initial learning [11, 57–59]. We examined this contrast across the whole brain, which revealed several clusters (S4 Table). The most prominent frontal region was the lPFC (Fig 3A), which partially overlapped with the lPFC region reported earlier. This effect was driven by a decrease in gray matter volume in NoDis Controls (F_1,9_ = 315.320, *p* < 0.001) and an increase in gray matter volume in Dis Controls (F_1,9_ = 127.39, *p* < 0.001). In addition, differences in gray matter were seen in part of the ACC/MCC and in cortex medial to the lOFC/12o in central OFC; the former also partially overlapped with the original ACC region. In summary, the results are in broad agreement with an argument that although DisRev may particularly tax certain cognitive processes, such as linking a choice to an outcome, the cognitive process is not unique to the reversal situation and is also required in the basic discrimination learning situation [11,57–59]. No changes, however, were linked to discrimination learning in the absence of any reversal component in the vicinity of plOFC/AI.

We also considered the possibility that gray matter may change not only as a function of the task performed in the experimental manipulation but also in a manner that was related to individual variation in performance. We therefore performed exploratory analyses to examine correlations between gray matter volume in the four frontal ROIs and performance metrics. We identified a significant negative relationship between lPFC gray matter in post-training scan 2 and the number of training days taken by the animals to reach criterion (r = −0.768, *p* = 0.016, Fig 3B). Notably, however, a similar pattern was present in the pretraining scan 1 (albeit at a nonsignificant level; r = −0.454, *p* = 0.219). Variation in lPFC, therefore, appears to relate to individual variation in the ability to perform the task. Such individual variation in lPFC may not be induced by individual variation in task learning, but rather, it might reflect individual variation that predicts learning. Such a result would be consistent with a very general role for lPFC in the learning process, perhaps through its involvement in top–down attentional processes [58].

Finally, we considered the possibility that there might be differences in the gray matter changes that occurred when animals learned choices that were defined by stimulus features in the object DisRev task or spatial position in the spatial DisRev task. A comparison between spatial and object DisRev learners revealed a significant session × area × group interaction (F_1,21_ = 5.80, *p* = 0.012). However, the effect is driven only by trends, with spatial learners showing a tendency toward less-pronounced gray matter changes in the plOFC/AI (F_1,7_ = 5.30, *p* = 0.055) and ACC/MCC (F_1,7_ = 4.09, *p* = 0.083) compared with object learners.

### Experiment 2

**Fig 4:**
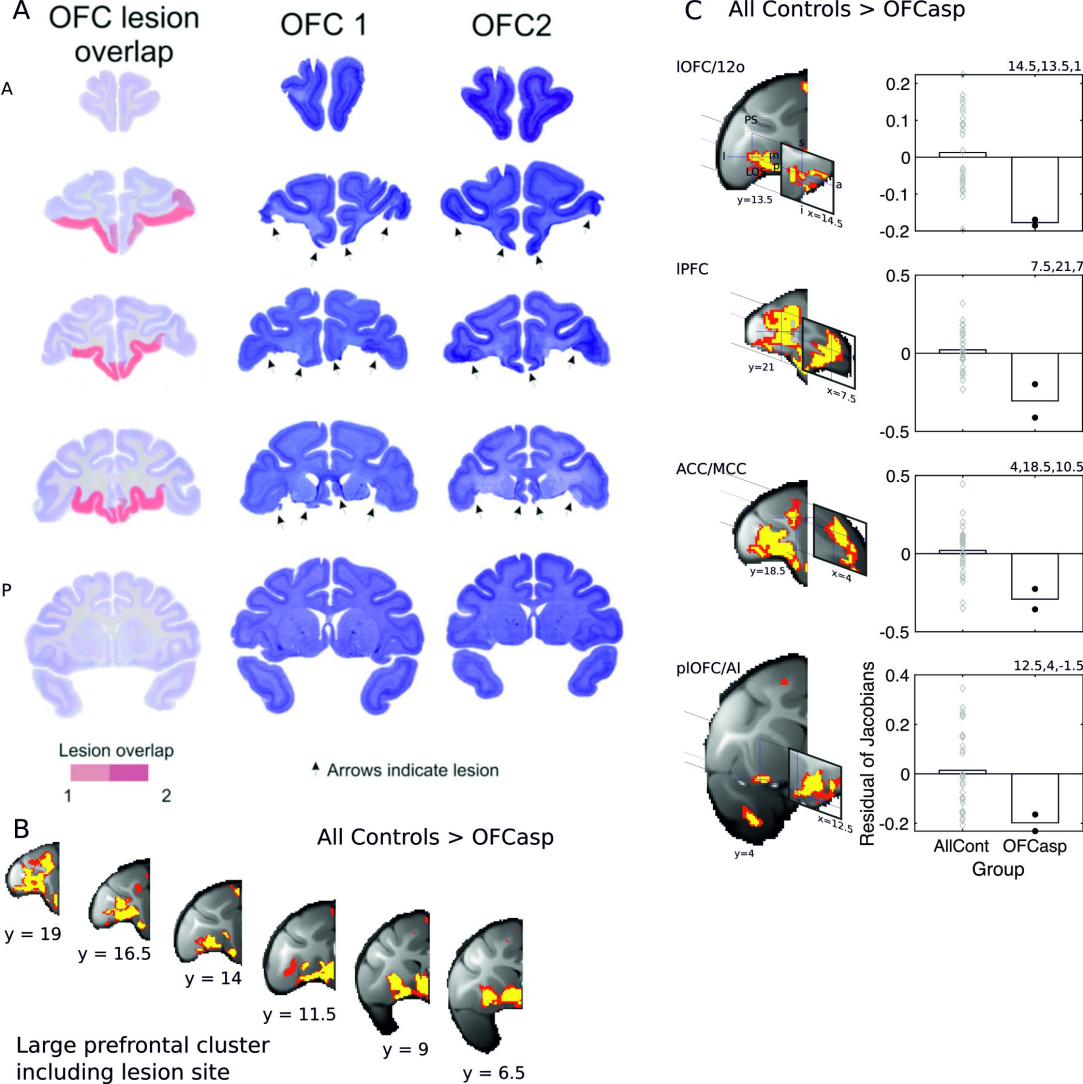
Lesions of central and medial OFC and adjacent vmPFC between the lateral orbital sulcus and the rostral sulcus. Panel A shows coronal sections through the frontal cortex (the most rostral section is shown at the top left, and the most caudal is shown on the bottom right). The lesions were intended to cover the same region investigated by Rudebeck and colleagues [3]. The actual lesion is indicated by red coloring. Saturated red color indicates area of lesion overlap in both animals, whereas paler red color indicates area of lesion in a single individual. Lesions were associated with large gray matter decrements in a distributed set of brain regions, including not only the aspiration lesion site itself (B) but also the four frontal regions associated with DisRev learning in experiment 1, including lOFC/12o (top row), lPFC on the ventral bank (second row), ACC/MCC, and plOFC/AI (C). In both panels B and C, the results illustrated are for bilaterally symmetrical effects (*p* < 0.001) and are shown in yellow and red (*p* < 0.005). Because in the initial stages of analysis, all scans from both before and after training are registered to a template derived from their average, the baseline residual Jacobian values in each figure lie close to the mean. 12o, orbital part of area 12; a, anterior; ACC/MCC, anterior cingulate cortex/midcingulate cortex; AllCont, all control; DisRev, discrimination reversal; l, lateral; lOFC, lateral OFC; lPFC, lateral prefrontal cortex; m, medial; OFC, orbitofrontal cortex; p, posterior; OFCasp, OFC aspiration lesion; plOFC, posterior lOFC; plOFC/AI, plOFC extending into AI; s, superior; vmPFC, ventromedial prefrontal cortex. Data are available on https://www.jeromesallet.org/data-ofc-reversal-learning and https://doi.org/10.5281/zenodo.3776631.

In order to investigate this network further and identify the wider structural network associated with central OFC aspiration lesions, we looked at the impact on gray matter across the brain of OFC lesions extending from the rostral sulcus on the medial surface of the frontal lobe to the medial bank of the lateral orbital sulcus in two macaques. The lesions included tissue sometimes described as vmPFC or mOFC [24,60] as well as the central parts of OFC (Fig 4A). However, they spared tissue lateral to the lateral orbitofrontal sulcus, implicated in linking choices and outcomes and the use of win–stay/lose–switch strategies during learning [9,14]. Thus, the lesion resembled those first made by Izquierdo and colleagues [61], which were described as large OFC aspiration lesions (as opposed to posterior OFC lesions) by Rudebeck and colleagues [3], and was focused on areas 14, 11, and 13 but spared area 12, including 12o [62,63]. We refer to the lesions as vmPFC/OFC lesions. We collected structural MRI scans from two macaques approximately 4 months after the aspiration lesions (Fig 4A) and compared them with structural MRIs from a control group of 28 control macaques (S1 Table). Note that the control group comprised some of the animals that would go on to learn the DisRev task in experiment 1, but all the scans examined were the ones taken at the first time point prior to any DisRev learning. The large control group made the DBM analysis sensitive to subtle changes in gray matter distant from the primary intended lesion site without requiring a large lesion group. The post-lesion delay in scanning was used to ensure that any neural changes were specific to the lesion intervention and not reflective of any general, immediate consequence of edema beyond the immediate lesion site that might occur during the immediate postsurgery recovery period. In addition to our regressor of interest (control versus lesion group), the age and sex of individual monkeys were included as control regressors in the GLM. As in experiment 1, the dependent variable was the determinant of the Jacobian matrix from the nonlinear registration of each individual’s structural MRI to the group average brain, and again, we sought regions in which effects were replicated in both hemispheres by testing for the conjoint probability of symmetrical effects in the two hemispheres with a *p* < 0.001 extended over more than 15 contiguous voxels (see Methods). We focused on regions of gray matter reduction in the lesion group relative to controls.

Aspiration lesions of vmPFC/OFC were associated with significant changes in gray matter in the frontal cortical regions identified in experiment 1: lOFC/12o, plOFC/AI, and ventral bank of the principal sulcus (lPFC) as well as the ACC/MCC (Fig 4B and 4C). Changes were also noticeable in some other areas (S5 Table).

For illustrative purposes, we present the average Jacobian values, after age and sex had been accounted for, in the lesion group and controls. Jacobian values were extracted from bilateral ROIs (3.375 mm^3^ in diameter) at coordinates reflecting the center of gravity of the bilateral cluster. In some cases, the effects were in relatively adjacent and large frontal clusters (lOFC/12o; lPFC). Therefore, to ensure that Jacobian values extracted from each ROI were independent of one another, they were taken from ROIs centered on coordinates just off the center of gravity of lOFC/12o and lPFC. Thus, each illustration reflects separate data as much as possible while still representing the anatomical regions. The precise coordinates of the ROI centers are all reported (S5 Table).

It might be worth noting that although both the lesion animals were female animals, it was still possible to include sex as a covariate in the comparison of the control and lesion groups because five of the control animals were females too. Although it was true that there was a nonsignificant tendency for male macaque brains to be bigger than female brains (t_26_ = 1.81, *p* = 0.0817), within the four frontal ROIs that are the focus of the investigation, residual gray matter (after partialling out the effect of animal weight) is significantly greater in control female brains than control male brains (F_1,26_ = 8.23, *p* = 0.008). Thus, any sex difference in gray matter in the four frontal ROIs runs counter to the gray matter effects we report in association with the lesion.

### Experiment 3

To further characterize the regions identified in experiments 1 and 2, we examined functional connectivity of the plOFC/AI where gray matter effects associated with DisRev training were greatest and most extensive in experiment 1 and where gray matter effects overlapped in experiments 1 and 2. The blood-oxygen-level-dependent (BOLD) signal was measured under anesthesia with fMRI. We examined whether pattern of coupling of the activity in plOFC/AI changed after DisRev learning (experiment 3) and after vmPFC/OFC lesions (experiment 4).

**Fig 5:**
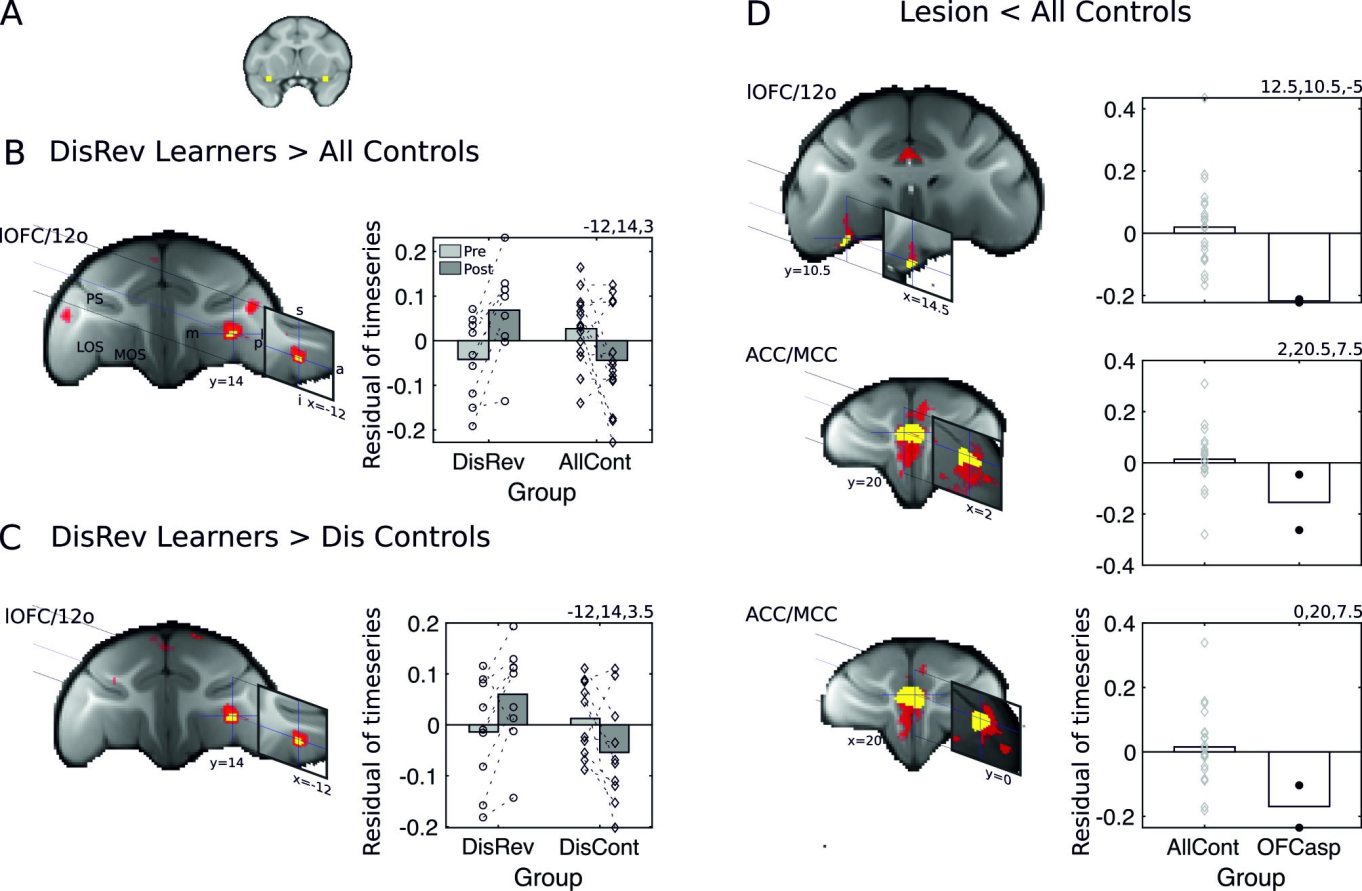
fMRI-measured activity coupling, at rest, between the brain regions identified in Figs 2 and 3. Learning DisRev, relative to all control animals, was associated with changes in functional coupling seeded bilaterally within the plOFC/AI (A) within some of the same set of brain regions as experiment 1: (B) plOFC/AI increased in functional coupling with the lOFC/12o. Results of tfce correction (*p* < 0.05) are shown in yellow and red (*p* < 0.01). (C) Similar results were found when comparing DisRev learners and the Dis Control group that had learned a discrimination learning task lacking a reversal component. (D) fMRI functional coupling of the plOFC/AI in the OFC aspiration lesion animals versus controls. Functional coupling between the plOFC/AI and lOFC/12o (top) and clusters in the left and right ACC/MCC identified in the ROI analysis (second and third rows). Across analyses, resting-state coupling shows relative increases after learning in the DisRev animals and relative decreases in the NoDis Controls or Dis Controls. 12o, orbital part of area 12; a, anterior; ACC/MCC, anterior cingulate cortex/midcingulate cortex; AI, anterior insula; AllCont, all control; Dis Control, discrimination control; DisRev, discrimination reversal; fMRI, functional magnetic resonance imaging; I, inferior; l, lateral; lOFC, lateral OFC; LOS, lateral orbital sulcus; m, medial; MOS, medial orbital sulcus; NoDis Control, no-discrimination control; OFC, orbitofrontal cortex; OFCasp, OFC aspiration lesion; p, posterior; plOFC, posterior lOFC; plOFC/AI, plOFC extending into AI; PS, principal sulcus; ROI, region of interest; s, superior; tfce, threshold-free cluster enhancement. Data are available on https://www.jeromesallet.org/data-ofc-reversal-learning and https://doi.org/10.5281/zenodo.3776631.

We examined the whole-brain functional connectivity of ROIs (15.625 mm^3^) in bilateral plOFC/AI (Fig 5A) using a seed-based correlation analyses in which the GLM design was equivalent to that described in experiment 1. However, rather than examining gray matter changes associated with DisRev learning, now the focus was on changes in the coupling of fMRI-measured activity. A within-subject, repeated measures design compared the functional connectivity of the bilateral plOFC/AI ROI (Fig 5A) at the time of scan 1 (prior to learning) and scan 2 (after learning) in DisRev animals and all control animals (*n* = 14). Resulting voxelwise p-maps were small volume cluster-corrected using threshold-free cluster enhancement methods [64] (*p* < 0.05) using large anatomical masks focused on the frontal regions identified across experiments 1 and 2; namely, lOFC/12o, lPFC, and ACC/MCC.

The analysis showed increased coupling between plOFC/AI and left lOFC/12o (Fig 5 and S6 Table). Paired *t* tests confirmed effects reflected both increases in activity coupling in DisRev learners (t_8_ = −3.52, *p* = 0.008) and decreases in controls (t_13_ = 2.33, *p* = 0.037). For illustration, we present the averaged residual time series, after controlling for age and gender, extracted from ROIs placed at the center of gravity of the significant cluster (3.375 mm^3^ in diameter) in the DisRev learners and all controls.

As in the DBM analysis, we sought to confirm that the changes in functional coupling were not dependent on nonspecific effects but were specific to the experience of reversing reward contingencies. We therefore examined the contrast between the DisRev learners and Dis Control learners (after excluding the NoDis Control animals). This more-selective analysis confirmed increased coupling between plOFC/AI and left lOFC/12o (Fig 5C and S6 Table). For illustration, we present the averaged residual time series, which is extracted from bilateral ROIs placed at the center of gravity of the significant clusters in the DisRev learners and Dis Controls.

### Experiment 4

Finally, we examined functional coupling of plOFC/AI in the lesion animals relative to controls (we considered here fMRI data collected at scan 1). Again, a seed-based correlation analysis focused on the same plOFC/AI ROI. The GLM used was equivalent to the one used to examine structural changes in experiment 2; it sought regions in which functional connectivity was reduced in lesion animals compared with controls. We report reduced coupling between plOFC/AI and lOFC/12o and bilateral ACC/MCC in the lesion group (Fig 5D and S6 Table). For illustration, we present the averaged residual time series, which was extracted from bilateral ROIs placed at the center of gravity of the significant clusters in the two lesion animals and the 22 control animals used in this analysis (although structural data were available for 28 control animals, fMRI data were only available for 22 animals).

Table 1 summarizes the DBM results (experiments 1 and 2) and resting-state fMRI results (experiments 3 and 4) from the learning (experiments 1 and 3) and lesion (experiments 2 and 4) investigations.

**Table 1:** Summary of the results (MNI coordinates and cluster size) across the four analyses (experiments 1 to 4) and two imaging methods (DBM or resting-state fMRI).

Analysis Type	DBM						Resting-State fMRI					
Contrast	DisRev > All Controls		DisRev > Dis Controls		Lesion < Controls*		DisRev > All Controls		DisRev > Dis Controls		Lesion < Controls	
Area	MNI (x, y, z)	Cluster (number of voxels)	MNI (x, y, z)	Cluster (number of voxels)	MNI (x, y, z)	Cluster (number of voxels)	MNI (x, y, z)	Cluster (number of voxels)	MNI (x, y, z)	Cluster (number of voxels)	MNI (x, y, z)	Cluster (number of voxels)
**lOFC/12o**	15.5, 14, 4	27	15.5, 14.5, 4	18	14.5, 13.5, 1	6,673	−12, 14, 3	17	−12, 14, 3.5	31	12.5, 10.5, −5	36
**lPFC**	16, 11, 8.5	110	17, 10, 8.5	70	7.5, 21, 7	6,673	-	-	-	-	-	-
**ACC/** **MCC**	2.5, 9, 9.5	86	3.5, 10.5, 10	75	4, 18.5, 10.5	6,673	-	-	-	-	2, 20.5, 7.5 0, 20, 7.5	184 298
**plOFC/AI**	14.5, 3, −7.5	256	14, 3.5, −6.5	309	12.5, 4, −1.5	6,673	-	-	-	-	-	-

*All frontal regions fall within a large cluster of lesion effects.

Abbreviations: 12o, orbital part of area 12; ACC/MCC, anterior cingulate cortex/midcingulate cortex; AI, anterior insula; DBM, deformation-based morphometry; Dis Control, discrimination control; DisRev, discrimination reversal; fMRI, functional magnetic resonance imaging; lOFC, lateral OFC; lPFC, lateral prefrontal cortex; MNI, Montreal neurological institute; OFC, orbitofrontal cortex; plOFC, posterior lOFC; plOFC/AI, plOFC extending into AI.

## Discussion

Learning to perform the DisRev task proficiently is associated with an extensive and bilateral region of gray matter change in plOFC/AI (experiment 1). Smaller regions of gray matter change were present in three other frontal cortical regions: lOFC/12o, ACC/MCC, and lPFC. However, notably, there were no changes in central OFC. These results complement recent claims that central OFC has only a limited role in DisRev [3,65]. Moreover, again in-line with the suggestion from Rudebeck and colleagues that aspiration lesions of central OFC disconnect the regions that are critical for reversal learning, we found that gray matter was reduced in all four of these areas when lesions were made in central OFC (experiment 2). This result is a likely consequence of damage caused to white matter fibers such as the uncinate fascicle, which runs close to the central OFC before branching out laterally to reach lateral prefrontal and cingulate regions. Experiment 3 confirmed that the way in which plOFC/AI interacted with other frontal cortical regions changed when animals learned the DisRev task. Moreover, the patterns of interaction found in experiment 3 were disrupted in experiment 4.

It would be possible to argue for a role of plOFC/AI and the other frontal areas in updating stimulus–outcome associations on the basis of the increased gray matter volume found when DisRev learners were compared with control subjects that did not learn any discrimination task. However, experiments 1 and 3 employed a second analysis in which DisRev learners were compared with control subjects trained to perform very similar two-option reward-guided visual discrimination tasks that lacked any reversal component. This made it possible to demonstrate that plOFC/AI is especially concerned with reversal learning. Behavioral adaptation in humans has also been associated with activity in AI [66,67]. The extensive effects found in plOFC/AI are also consistent with a recent finding that activity in the same region carries a signal that reflects not just whether a choice is rewarded but also the average level of reward received regardless of which choice is taken [68]. It is precisely these two quantities, the history of rewards for specific choices and the average level of rewards regardless of specific choices, which must be considered and balanced in a very particular manner when animals learn DisRev. Unlike in many natural environments, in DisRev, animals must learn to assign more prominence to specific choice–reward associations at the expense of the general reward history.

The co-recruitment of plOFC/AI and another region near to, but outside, central OFC, lOFC/12o, may reflect direct connections between the areas [69,70]. In rat, pharmacological lesions of the lateral orbital and AI are associated with reversal learning deficits [20]. Future studies will aim at disentangling the specific roles of the two regions in primates—plOFC/AI and lOFC/12o—closest to central OFC in promoting behavioral flexibility. It is also possible to link the present results with fMRI activity recorded in a nearby region when monkeys learn to use specific choice–reward outcomes to guide decision-making [9] and reversal learning or task switching in humans [71,72], reinforcing the idea of a similar structural–functional organization of the OFC and its subdivisions in macaques and humans [73]. Lesions that include this OFC region disrupt the process of reward credit assignment to specific choices [14], whereas AI reversible lesions affect retrieval of goal value to guide decisions [21].

Considerable effort has been dedicated to identifying the specific contributions of OFC to behavior [1–4,14,58,74]. It is becoming increasingly clear that its role can only be understood if allowance is made for functional heterogeneity within it. For example, activity in a reward-guided task varies across different OFC subdivisions [75], and the central OFC appears to have a particularly important role in determining the identity and desirability of rewards and other aspects of reward-guided decision-making assessed by devaluation tasks. By contrast, the present results suggest that fluent DisRev performance is mediated by interactions between plOFC/AI and lOFC/12o. In rat, functional heterogeneity of the OFC has also been reported [20,76,77].

Finally, our results raise the possibility that interactions with other areas within the DisRev network, including ACC/MCC and lPFC, are also important. These two regions have been previously associated with reversal learning [14,78]. ACC/MCC holds information about the value of choices other than the one that is being taken right now and translates such counterfactual choice values into actual behavioral change and exploration [30,31,79,80]. DisRev may also be mediated by the acquisition of cognitive sets or task models that are dependent on lPFC and its interactions with other brain regions. Such interactions may be needed to represent the interrelationships between the different choice–reward associations active in the task at different times [81,82]. It is possible that AI is also associated with such processes [83]. Our results demonstrate gray matter changes in ACC/MCC, lOFC/12o, and lPFC areas that are significantly greater when animals learn DisRev tasks. However, although the cognitive processes with which the areas are linked may be particularly taxed when constant relearning or task set selection is needed during DisRev, it is likely that the same processes are called into play during initial learning [14,24,57,59,84].

Beyond common circuits, previous results have also highlighted differences between object and spatial reversal learning [85–87], although the strength of such differences has also been questioned [59, 88]. Our direct comparison of object versus spatial reversal learners only revealed trends toward differences in effects, but these were broadly consistent with previous claims that a region within OFC, such as the plOFC, is more important for object-based reversal learning.

In addition to aiding identification of the neural circuit mediating behavioral flexibility, the present results have more general implications. Individual variation in prefrontal circuits has been linked to variation in a wide variety of behavioral measures, including general intelligence and lifestyle demographics on the one hand and psychiatric illnesses on the other hand [89,90]. The present results, however, suggest that interindividual variability in circuits comprising prefrontal components may not only have an endogenous source. Instead, they may also reflect differences in experience that have placed varying demands on cognitive mechanisms in different subjects. Just as the structural changes in our macaques were correlated with improved ability to negotiate the challenges of DisRev, so too the neural variation in healthy humans and patients may also be associated with variation in the ability to deal with new cognitive challenges.

## Methods

### Ethics statement

All procedures were conducted under licenses from the United Kingdom Home Office in accordance with the Animals (Scientific Procedures) Act 1986 and the European Union guidelines (EU Directive 2010/63/EU).

### Subjects

In total, 30 animals (seven females) were involved in the study (S1 Table).

### Experiment 1: DisRev learning

Four animals (OB1–4) learned an object-based DisRev task (object DisRev), five animals (SB1–5) learned a spatial-based DisRev task (spatial DisRev), and a total of 20 (control) animals acted as controls (C1–20). See S1 Table for details. Half of the 20 control animals (C1–10) had no experience of formal training (NoDis Control), whereas the other half had experience of performing two-option reward-guided visual discrimination tasks (Dis Control). Crucially, however, no controls had experience of DisRev. Two MRI scans were acquired in all animals, one prior to DisRev training and one after the learning criterion had been met (see Methods). Data from all of these animals were analyzed in a DBM analysis of brain structural changes associated with DisRev learning. Of these 20 control animals, 14 animals were included as controls in the fMRI analysis. This subset was chosen because they had all received the same isoflurane anesthetic agent as the experimental animals, OB1–4 and SB1–5; the anesthetic agent can impact on fMRI analyses, although it does not impact on DBM structural analyses.

### Experiment 2: Central and mOFC aspiration lesion

Two animals received lesions of central and mOFC and adjacent vmPFC (OFC1 and OFC2 were referred to as vmPFC/OFC lesions; Fig 4A), and a comparison of brain structure and functional activity coupling was made between them and a control group (*n* = 28 for structural analysis, and *n* = 22 for fMRI analysis). In total, 30 macaques were included in the DBM analysis of brain structural changes, and 24 macaques were included in the resting fMRI analysis (again, it was not possible to include all animals in the fMRI analysis). S1 Table details demographic information for the aspiration lesion and control animals used in experiment 2. The control animals were OB1–4, SB1–5, C1–10, and C12–20. Both control group and lesion group scans were obtained prior to any learning of DisRev tasks.

### Training histories

The nine animals in the reversal learning groups (OB1–4, SB1–5) were trained on the following protocol. Animals were initially trained to touch a blue target on screen. The target stimulus appeared either on the left or right of the screen in blocks of 50 trials for a total of 100 trials. The animals had their first scan once they had reached a performance criterion of 90%.

After the first scan, the training regime introduced target-based DisRevs in an incremental manner. Analogous procedures were used for both the object choice and spatial choice tasks (object DisRev and spatial DisRev). In both cases, the tasks were deterministic; at any given time, one choice was rewarded, and the other choice was not rewarded. The first testing schedule, schedule 1 (Fig 1), primed the rewarded choice for 25 trials before introducing, in addition, the unrewarded choice on the opposite side of the screen for an additional 75 trials. The choice–reward contingencies were reversed after the animals performed above 85% correct for two consecutive days. The animals experienced two choice–reward reversals under this protocol. The second testing schedule, schedule 2, followed the same pattern, but now the rewarded choice was not primed. Animals completed 100 trials, in which one of two simultaneously presented options was designated as the rewarded target for the day. Again, contingencies reversed after two consecutive days of 85% correct performance. Animals experienced a total of five reversals under this protocol. With perfect performance, an animal could, therefore, complete this second phase in 10 days. Schedules 3 and 4 introduced a choice–reward association reversal within a given day’s testing session; the reversal occurred once the animal had correctly chosen the target 50 times. The animals completed two days of 100 trials and three days of 150 trials on schedules 3 and 4, respectively. There was no performance criterion in these phases. In the final schedule, the rewarded targets reversed after 25 correctly performed trials. In total, the animals had to perform 150 correct trials in a day. Once the animals’ performance was over 80% for two consecutive days, with a subsequent 12 days of consolidation training, their second scan was taken.

The training experience of the 20 control monkeys varied within the group but, critically, did not include choice–reward reversal learning. Four animals had been trained on a fixation task. Six animals were involved in a neuroanatomy study, so they had no formal training between their two scans [91]. These 10 animals are referred to as the NoDis Controls. Another 10 animals, Dis Controls, learned to discriminate between rewarded objects but had not experienced choice–reward reversals. No surgical intervention occurred between the two scans.

Note that in the first two experiments, two animals (lesion 1, lesion 2) received lesions of the central and mOFC and adjacent vmPFC (referred to as a vmPFC/OFC lesion). Prior to their lesion, these animals had learned to discriminate between objects but had no experience of choice–reward reversals.

### Apparatus

Animals were trained via positive reinforcement to stay inside an MRI-compatible chair in a sphinx position that was placed inside a homemade mock scanner that simulated the MRI scanning environment. They made responses on a touch-sensitive monitor (38 cm wide × 28 cm high) in front of them, on which visual stimuli could be presented (eight-bit color clip art bitmap images, 128 × 128 pixels).

Smoothie rewards (banana mixed with diluted black currant squash) were delivered from a spout immediately in front of the animal. At the end of testing, the animals were given their daily food allowance, consisting of proprietary monkey food, fruit, peanuts, and seeds, delivered immediately after testing each day. This food was supplemented by a forage mix of seeds and grains given approximately 6 hours before testing in the home cage. Stimulus presentation, experimental contingencies, and reward delivery were controlled by a computer using in-house programs.

### Lesion surgery

At least 12 hours before surgery, macaques were treated with an antibiotic (8.75 mg/kg amoxicillin, intramuscularly [i.m.]) and a steroidal anti-inflammatory (20 mg/kg methylprednisolone, i.m.) to reduce the risk of postoperative infection, edema, and inflammation. Additional supplements of steroids were given at 4- to 6-hour intervals during surgery. On the morning of surgery, animals were sedated with ketamine (10 mg/kg, i.m.) and xylazine (0.5 mg/kg, i.m.) and given injections of atropine (0.05 mg/kg), an opioid (0.01 mg/kg buprenorphine), and a nonsteroidal anti-inflammatory (0.2 mg/kg meloxicam) to reduce secretions and provide analgesia, respectively. The monkeys were also treated with an H2 receptor antagonist (1 mg/kg ranitidine) to protect against gastric ulceration, which might have occurred as a result of administering both steroid and nonsteroidal anti-inflammatory treatments. Macaques were then moved to the operating theater, where they were intubated, switched onto sevoflurane anesthesia, and placed in a head holder. The head was shaved and cleaned using antimicrobial scrub and alcohol.

A midline incision was made, the tissue was retracted in anatomical layers, and a bilateral bone flap was removed. All lesions were made by aspiration with a fine-gauge sucker. Throughout the surgery, heart rate, respiration rate, blood pressure, expired CO_2_, and body temperature were continuously monitored. At the completion of the lesion, the wound was closed in anatomical layers. Nonsteroidal anti-inflammatory analgesic (0.2 mg/kg meloxicam, orally) and antibiotic (8.75 mg/kg amoxicillin, orally) treatment was administered for at least 5 days postoperatively. All surgery was carried out under sterile conditions with the aid of a binocular microscope. Aspiration lesions of the central and mOFC/vmPFC were intended to resemble the aspiration lesions that Rudebeck and colleagues [3] found disrupted DisRev task performance; they were therefore placed between the lateral orbitofrontal sulcus and the rostral sulcus (predominantly Walker’s areas 11, 13, and 14).

Approximately 4 months later, the animals were scanned under anesthesia. Using the same protocol as described below (which was the same for control animals), we collected structural and resting-state images.

### MRI data collection

Protocols for animal care, MRI, and anesthesia were similar to those that we have previously described [48, 49]. During scanning, under veterinary advice, animals were kept under minimum anesthetic levels using isoflurane. A four-channel phased-array coil was used (Windmiller Kolster Scientific, Fresno, CA). Structural scans were acquired using a T1-weighted MPRAGE sequence (no slice gap, 0.5 × 0.5 × 0.5 mm, TR = 2,500 milliseconds, TE = 4.01 milliseconds, 128 slices). Whole-brain BOLD fMRI data were collected for 53 minutes, 26 seconds from each animal, using the following parameters: 36 axial slices, in-plane resolution 262 mm, slice thickness 2 mm, no slice gap, TR = 2,000 milliseconds, TE = 19 milliseconds, 1,600 volumes.

### DBM analysis of structural MRI data

The custom-made scripts used for our DBM analysis uses the FSL linear and nonlinear transformation tools FLIRT and FNIRT. The logic of the approach is that if a group of brain images can be warped to a near-identical image, then differences in that warping process give a measure of the local differences in brain structure between individuals. DBM has been previously used in several studies on brain plasticity [38,39,42,48–50]. All the brains were first aligned to the MNI rhesus macaque atlas template [92,93] using the affine registration tool FLIRT [94,95] followed by nonlinear registration using FNIRT [96], which uses a b-spline representation of the registration warp field [97]. The resulting images were averaged to create a study-specific template. This process underwent five iterations, each time increasing the resolution of the warps and refining the template. The final iteration was performed with a warp resolution (knot-spacing of cubic B-splines) of 1 mm. At this final stage, the brains from the different monkeys were almost indistinguishable to an observer. The determinant of the Jacobian of the warp field was then extracted—the Jacobian is a matrix of the directional stretches required to register one image to another, and the determinant of this matrix gives a scalar value for the volumetric change implied. The Jacobian values were then used as the dependent variable in the statistical analyses of reversal learning (experiment 1) or the effects of the central OFC and mOFC/vmPFC aspiration lesion (experiment 2). To avoid potential misalignment concerns, in experiment 2, the lesion animals were not used to create the study-specific template (see S1 Fig for MRI gray matter coronal sections of lesion animals aligned to study-specific template). In experiment 1, all animals (learners and controls) were included.

GLM analyses were adapted from Winkler and colleagues [51], allowing, when necessary, permutation inference analysis with our repeated measure experimental design (two structural scans collected during each of the two scan periods [pre- and post-learning] per subject). In addition to our regressor of interest (behavioral condition in experiment 1: all learners versus all controls or control versus lesion group in experiment 2; see S2 Fig for graphical representation of the statistical analysis), we also included control regressors indexing age and sex of individual monkeys. We implemented the nonparametric Randomise procedure to perform permutation-based nonparametric testing, examining positive and negative contrasts. The approach was used to identify brain regions that were larger at scan 2 compared with scan 1 in the DisRev learning groups (object DisRev and spatial DisRev) compared with Dis Controls only. In experiment 2, we focus on gray matter decrements related to the lesion. Permutation tests for the lesion scans were performed in the same manner as for the reversal learning experiment, with the exception that Randomise automatically runs fewer permutations because of the smaller sample size.

The originators of MRI voxel-based gray matter analyses emphasized that taking into account the spatial extent across adjacent MRI voxels of any statistical effect may not be appropriate for gray matter analyses [53]. Instead, an alternative test of robustness involves examining whether effects are bilaterally symmetrical [49,53–55]. The premise rests on the assumption that if a statistical effect noted had a chance of occurrence of *p* < 0.032 in one brain area under the null hypothesis, then it has the chance of occurring in the same area in both hemispheres with the square of this probability (i.e., *p* < 0.001). Therefore, to identify gray matter changes that were bilateral, we applied an uncorrected threshold of *p* < 0.032 to both hemispheres and binarized the thresholded image. The right hemisphere image was then dimensionally transformed along the x-axis (around the midsagittal plane) and linearly registered with the left hemisphere using FLIRT. The two images were then multiplied, allowing us to examine gray matter in areas in which effect significance was *p* < 0.001 and extended over 15 voxels (corresponding to 1.125 mm^3^).

For illustrative purposes, Jacobian values were extracted for each structural scan from binarized 3-mm^3^ cube masks placed at the center of gravity of the effect clusters (*p* = 0.001) in both hemispheres. In experiment 1, we present the Jacobian values in the two groups at scan times 1 and 2. In experiment 2, we simply present the Jacobian values in the lesion and control groups separately. The residual variance in Jacobian values, after sex and age were accounted for, was then averaged across hemisphere and structural scans. To represent variance, we index Jacobian values for each contributing animal.

### fMRI analysis of activity coupling

Prior to fMRI analysis, the following preprocessing was applied: removal of nonbrain voxels, discarding of the first six volumes of each fMRI dataset, 0.1-Hz low-pass filtering to remove respiratory artifacts, motion correction, spatial smoothing (Gaussian 3-mm FWHM kernel), grand-mean intensity normalization of the entire 4D dataset by a single multiplicative factor, high-pass temporal filtering (Gaussian-weighted least-squares straight line fitting, with sigma = 50.0 seconds). Registration of functional images to the skull-stripped structural MRI scan and to the MNI macaque template [92,93] was achieved with linear and nonlinear registration using FLIRT and FNIRT, respectively [94].

To establish changes in functional connectivity within the network as a function of DisRev learning (experiment 1) or lesions (experiment 2), we used a voxel-wise whole-brain approach to map resting-state functional connectivity of the plOFC/AI. The 15.6-mm^3^ cube ROIs were made at the center of the gray matter cluster identified in the DBM analyses in experiment 2, displaced minimally to reduce white matter inclusion. The binary images were registered to each monkey’s fMRI scan. The BOLD time series was then extracted from each ROI in each individual animal. Analyses were performed using tools from FSL [98] using the method described by Mars and colleagues [99].

For each animal, first, we calculated the first eigen time series of the BOLD signal in the ROI. The first eigen time series is the single time series that best reflects coherent activity across the ROI in that it represents the largest amount of variance across the set of voxels within the region. At the individual subject level, we fitted a GLM consisting of the first eigen time series and seven confound regressors—namely, the average time series of the whole brain and six movement parameters expressing movement during the scan as calculated using the FSL tool MCFLIRT (note that the scans were obtained from anesthetized animals while their heads were fixed in a stereotaxic frame).

### Experiment 3: DisRev learning

The resting-state analyses were equivalent to those described for the structural-based experiments. A GLM was created with a repeated measure experimental design (fMRI scan [scan 1 and scan 2] per subject). In addition to our regression of interest for this analysis (all learners [*n* = 9] versus all controls [*n* = 14]), we also included control regressors indexing age and sex of individual monkeys. Randomise was implemented to perform permutation-based nonparametric testing, examining positive contrasts. Resulting voxelwise p-maps were small volumes cluster-corrected using threshold-free cluster enhancement methods (*p* < 0.05) using anatomical masks focused on the frontal regions identified across experiments 1 and 2; namely, lOFC/12o (left = 1,235 mm^3^, right = 1,187.75 mm^3^), lPFC (left = 674.25 mm^3^, right = 614 mm^3^), and ACC/MCC (left = 1,222.25 mm^3^, right = 100.75 mm^3^).

The same approach was used to identify brain regions that were larger at scan 2 compared with scan 1 in the DisRev learning animals compared with Dis Controls only.

### Experiment 4: Central and mOFC aspiration lesion

Finally, in experiment 4, we examined plOFC/AI functional connectivity in lesion animals (*n* = 2) compared with all controls (*n* = 22) from their post-lesion or prelearning scans, respectively, and again sought a between-group difference with reduced connectivity in lesion compared with control animals.

For illustrative purposes, we present the averaged residual functional connectivity values following the same procedure described for the DMN analysis. For all analyses, functional connectivity values were extracted from binarized 3-mm^3^ cube masks placed at the center of gravity of the effect clusters in significant hemispheres. The residual variance in functional connectivity values, after sex and age were accounted for, was then averaged across hemispheres. To represent variance, we index functional connectivity values for each contributing animal.

### Data, scripts, and software availability

FSL can be downloaded from https://fsl.fmrib.ox.ac.uk/fsl/fslwiki.

All dedicated scripts and data have been deposited online (https://www.jeromesallet.org/data-ofc-reversal-learning and https://doi.org/10.5281/zenodo.3776631).

## Supporting information

S1 Fig Native gray matter structural scans are nonlinearly registered to the study-specific template. Lesion animals (OFC1 and OFC2) are shown, with the extent of the lesion indicated with white arrows. Two exemplar control animals are shown for comparison: a female (C5) and male (C13) representing the medium brain sizes of their respective sexes. A, anterior; C, control; OFC, orbitofrontal cortex; P, posterior.
(EPS)

S2 Fig Visualisation of GLM used for the OFC lesion versus control subjects comparison. GLM, general linear model; OFC, orbitofrontal cortex.
(TIFF)

S3 Fig A 3D-rendered illustration of the DisRev learners > all controls contrast from experiment 1. AI, anterior insula; DisRev, discrimination reversal; lOFC, lateral OFC; lPFC, lateral prefrontal cortex; OFC, orbitofrontal cortex; PS, principal sulcus.
(EPS)

S1 Table List of subjects involved in the present study. (DOCX)

S2 Table Structural changes associated with DisRev learning from the comparison DisRev learners versus all controls (experiment 1) scan 2 > scan 1. DisRev, discrimination reversal.
(DOCX)

S3 Table Structural changes associated with DisRev learning from the comparison DisRev learners versus Dis Controls (experiment 1) scan 2 > scan 1. Dis Control, discrimination control; DisRev, discrimination reversal.
(DOCX)

S4 Table Structural changes associated with DisRev learning from the comparison Dis Controls > NoDis Controls (experiment 1, supplementary contrast) scan 2 > scan 1. Dis Control, discrimination control; DisRev, discrimination reversal; NoDis Control, no-discrimination control.
(DOCX)

S5 Table Structural changes associated with OFC lesions (experiment 2). OFC, orbitofrontal cortex.
(DOCX)

S6 Table Functional changes associated with OFC lesions and discrimination reversal learning (experiment 3 and 4). OFC, orbitofrontal cortex.
(DOCX)

## Abbreviations

12o: orbital part of area 12
ACC: anterior part of cingulate cortex
AI: anterior insula
BOLD: blood-oxygen-level-dependent
DBM: deformation-based morphometry
Dis Control: discrimination control
DisRev: discrimination reversal
fMRI: functional MRI
FMRIB: Oxford Centre for Functional Magnetic Resonance Imaging
FSL: FMRIB Software Library
GLM: general linear model
ITI: intertrial interval
lOFC: lateral OFC
LOS: lateral orbital sulcus
lPFC: lateral prefrontal cortex
MCC: midcingulate cortex
MNI: Montreal neurological institute
mOFC: medial OFC
MOS: medial orbital sulcus
MRI: magnetic resonance imaging
NoDis Control: no-discrimination control
OFC: orbitofrontal cortex
plOFC: posterior lOFC
plOFC/AI: plOFC extending into AI
ROI: region of interest
vmPFC: ventromedial prefrontal cortex.
