# Supplementary figures and images for: Behavioral flexibility is associated with changes in structure and function distributed across a frontal cortical network in macaques
Source: PLoS Biol. 2020 May 26;18(5):e3000605. doi: 10.1371/journal.pbio.3000605 (PMC7274449; doi:10.1371/journal.pbio.3000605)

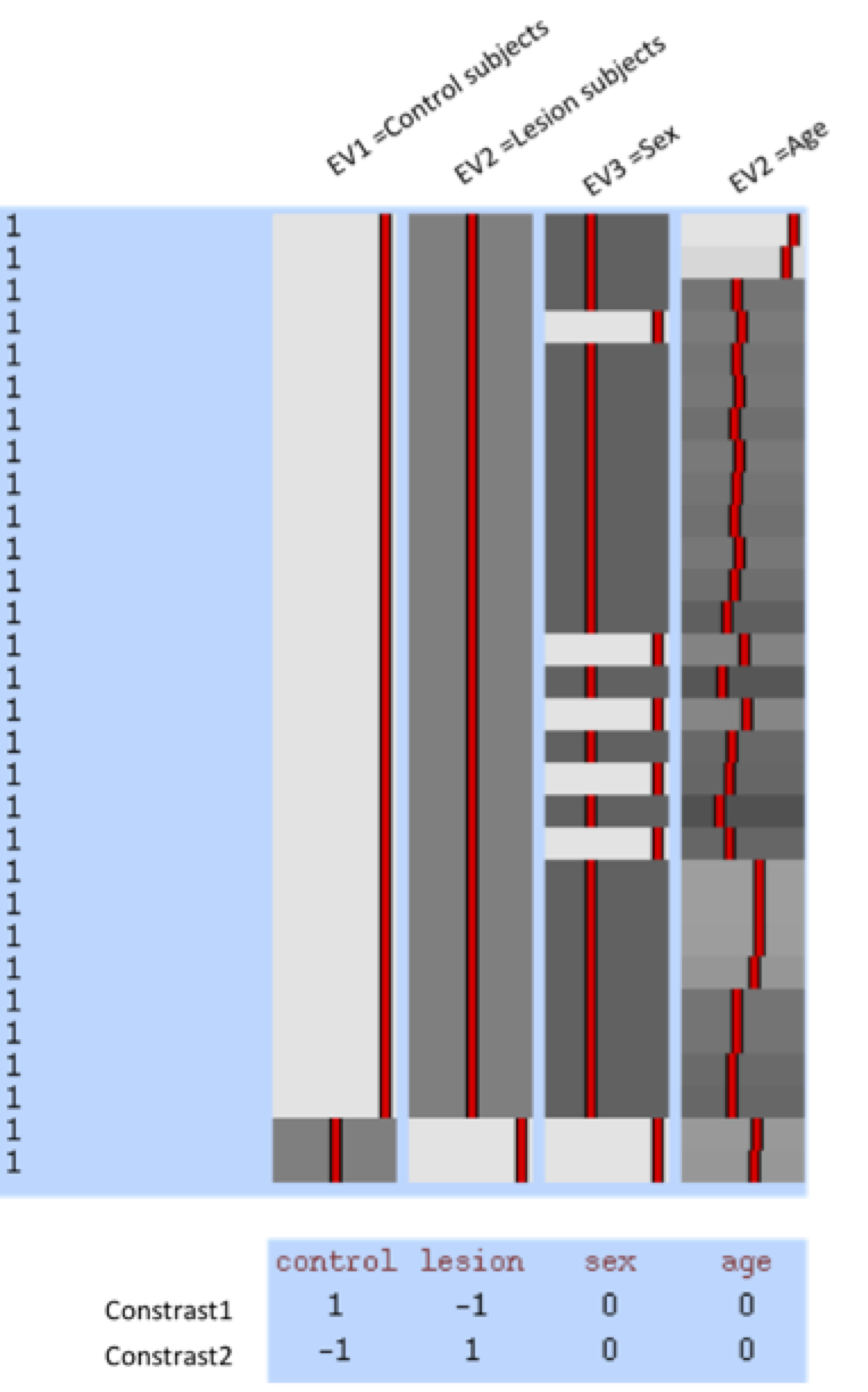

Supplement: S2 Fig — GLM, general linear model; OFC, orbitofrontal cortex. (TIFF) [file pbio.3000605.s002.tiff]
