# Supplementary material for: Behavioral flexibility is associated with changes in structure and function distributed across a frontal cortical network in macaques
Source: PLoS Biol. 2020 May 26;18(5):e3000605. doi: 10.1371/journal.pbio.3000605 (PMC7274449; doi:10.1371/journal.pbio.3000605)
Supplement: S1 Table — (DOCX) [file pbio.3000605.s004.docx]

**Subjects**

In total 30 animals (seven females) were involved in the study (Table S1).

**S1 Table**

| Animal | Group | Learning History | Age Scan1 | Age Scan2 | Age Scan3 | Sex |
| --- | --- | --- | --- | --- | --- | --- |
| OB1 | Object DisRev |  | 4.36 | 4.95 | 5.14 | M |
| OB2 | Object DisRev |  | 3.93 | 5.03 | 5.16 | M |
| OB3 | Object DisRev |  | 4.05 | 4.97 | 5.10 | M |
| OB4 | Object DisRev |  | 4.54 | 5.23 | 5.34 | M |
| SB1 | Spatial DisRev |  | 4.40 | 5.14 | 5.39 | M |
| SB2 | Spatial DisRev |  | 4.39 | 5.01 | 5.21 | M |
| SB3 | Spatial DisRev |  | 4.58 | 4.87 | 5.02 | M |
| SB4 | Spatial DisRev |  | 4.69 | 5.06 | 5.29 | M |
| SB5 | Spatial DisRev |  | 4.01 | 5.08 | 5.29 | M |
| C1† | NoDis Control | no task learned | 11.21 | 11.86 | 11.94 | M |
| C2† | NoDis Control | no task learned | 10.47 | 10.93 | 11.07 | M |
| C3 | NoDis Control | visual fixation task | 3.04 | 4.33 |  | M |
| C4† | NoDis Control | no task learned | 5.49 | 5.86 | 5.90 | F |
| C5† | NoDis Control | no task learned | 5.31 | 5.68 | 5.72 | F |
| C6 | NoDis Control | visual fixation task | 3.60 | 4.84 |  | M |
| C7 | NoDis Control | visual fixation task | 2.41 | 4.71 |  | M |
| C8† | NoDis Control | visual fixation task | 3.44 | 4.05 | 4.38 | M |
| C9 | NoDis Control | no task learned | 2.08 | 4.38 |  | F |
| C10† | NoDis Control | no task learned | 3.44 | 4.06 | 4.41 | F |
| C11* | Dis Control | target discrimination task | 3.79 | 6.05 |  | F |
| C12 | Dis Control | target discrimination task | 4.79 | 5.06 |  | F |
| C13 | Dis Control | target discrimination task | 6.97 | 7.27 |  | M |
| C14 | Dis Control | target discrimination task | 6.99 | 7.38 |  | M |
| C15 | Dis Control | target discrimination task | 6.90 | 7.30 |  | M |
| C16 | Dis Control | target discrimination task | 6.49 | 6.86 |  | M |
| C17 | Control | target discrimination task | 4.37 | 4.77 |  | M |
| C18 | Dis Control | target discrimination task | 4.39 | 4.74 | 4.99 | M |
| C19 | Dis Control | target discrimination task | 3.71 | 4.11 | 4.44 | M |
| C20 | Dis Control | target discrimination task | 3.50 | 3.74 |  | M |
| OFC1* | OFC/vmPFC lesion | target discrimination task | 6.05 | 6.55 |  | F |
| OFC2 | OFC/vmPFC lesion | target discrimination task | 6.15 | 6.69 |  | F |

*C11 and OFC1 are the same animal. The data identified as C11 is the animal’s pre-operative scan. While the data from this animal could be used for control purposes in the longitudinal experiment 2 it could not be used as control data in the between-subject experimental design employed in the lesion experiments. †Monkeys scanned with sevoflurane and not isoflurane were not used in the fMRI analysis in experiment 1 or 2 but only in the DBM structural MRI analysis. While the type of anesthetic used may impact on fMRI data it does not impact on structural MRI data.
