# Supplementary material for: Behavioral flexibility is associated with changes in structure and function distributed across a frontal cortical network in macaques
Source: PLoS Biol. 2020 May 26;18(5):e3000605. doi: 10.1371/journal.pbio.3000605 (PMC7274449; doi:10.1371/journal.pbio.3000605)
Supplement: S2 Table — DisRev, discrimination reversal. (DOCX) [file pbio.3000605.s005.docx]

**Structural changes associated with discrimination reversal learning**

**S2 Table : DBM results table: DisRev Learners > All Controls (experiment 1) Scan 2 > Scan 1**

| Region | x | y | x | Cluster extent (num vox) p < 0.001 |
| --- | --- | --- | --- | --- |
| lOFC (12o) | 15.5 | 14 | 4 | 27 |
| lPFC (46v) | 16 | 11 | 8.5 | 110 |
| IPFC (46d) | 13 | 9.5 | 12 | 54 |
| ACC/MCC (24c) | 6 | 12 | 10.5 | 25 |
| ACC/MCC (24a) | 2.5 | 9 | 9.5 | 86 |
| plOFC/AI | 14.5 | 3 | -7.5 | 256 |
| Striatum (caudate) | 6 | 8 | 7 | 26 |
| Striatum (caudate) | 4.5 | 1 | 8.5 | 30 |
| Striatum (putamen) | 9.5 | 4.5 | 0.5 | 42 |
| Basal Forebrain (Basal Nucleus of Meynert) | 4 | 2.5 | -4.5 | 116 |
| Inferotemporal cortex (TE) | 16.5 | 0.5 | -14.5 | 26 |
| Inferotemporal cortex (TEO) | 27 | -13.5 | 3 | 45 |
| Posterior STS (Tpt) | 21.5 | -16 | 9 | 21 |
| Amygdala (ABmc) | 7.5 | 0 | -7.5 | 87 |
| Hippocampus | 7.5 | -4.5 | -9 | 48 |
| Substantia Nigra | 4.5 | -10 | -8 | 28 |
| Parietal cortex (7b) | 20 | -13.5 | 13.5 | 75 |
| Occipital Cortex (V4) | 14 | -19.5 | -3 | 29 |
|  | 18.5 | -24.5 | -5.5 | 26 |
| Occipital Cortex (V4) | 22.5 | -25 | 8 | 83 |
|  | 26 | -24.5 | 3.5 | 45 |
|  | 25 | -24.5 | -4.5 | 71 |
| Occipital Cortex (V3) | 19.5 | -28.5 | -6.5 | 146 |
|  | 9.5 | -31 | 17 | 1995 |
| Occipital Cortex (V2) | 26 | -24 | 0.5 | 15 |
|  | 8.5 | -29.5 | 12 | 43 |
|  | 5.5 | -31.5 | 4.5 | 33 |
|  | 7 | -28.5 | 3 | 35 |
| Occipital Cortex (V1) | 25.5 | -28 | -0.5 | 82 |
|  | 25 | -29.5 | 1.5 | 39 |
|  | 23 | -32.5 | -0.5 | 18 |
| Cerebellum | 10.5 | -26.5 | -1.5 | 23 |
|  | 2.5 | -24 | -0.5 | 85 |
|  | 7.5 | -32 | -1 | 49 |
