# Supplementary material for: Behavioral flexibility is associated with changes in structure and function distributed across a frontal cortical network in macaques
Source: PLoS Biol. 2020 May 26;18(5):e3000605. doi: 10.1371/journal.pbio.3000605 (PMC7274449; doi:10.1371/journal.pbio.3000605)
Supplement: S3 Table — Dis Control, discrimination control; DisRev, discrimination reversal. (DOCX) [file pbio.3000605.s006.docx]

**S3 Table : DBM results table: DisRev Learners > Dis Controls (experiment 1) Scan 2 > Scan 1**

| Region | x | y | z | Cluster extent (num vox) p < 0.001 |
| --- | --- | --- | --- | --- |
| mOFC (14r) | 2 | 18.5 | -1.5 | 20 |
| lOFC (12o) | 15.5 | 14.5 | 4 | 18 |
| lPFC (46v) | 18.5 | 14.5 | 7.5 | 15 |
| lPFC (8Ad/v) | 17 | 10 | 8.5 | 70 |
| ACC/MCC (24c) | 6 | 16 | 12 | 20 |
| ACC/MCC (24c) | 5.5 | 15 | 9.5 | 45 |
| ACC/MCC (24b) | 3.5 | 10.5 | 10 | 75 |
| plOFC/AI | 14 | 3.5 | -6.5 | 309 |
| Premotor Cortex (F2) | 7 | 9.5 | 18 | 16 |
| Striatum (caudate) | 5.5 | 7.5 | 6.5 | 55 |
| Striatum (caudate) | 5 | 2 | 8.5 | 83 |
| Basal Forebrain (Nucleus Basalis of Meynert) | 3.5 | 1.5 | -5 | 31 |
| Striatum (putamen) | 10 | 3 | -0.5 | 136 |
| Insula (Id) | 19 | 0.5 | -1.5 | 21 |
| Inferotemporal Cortex (TGa) | 16.5 | 1.5 | -15 | 102 |
| Entorhinal cortex | 9 | -3 | -14.5 | 18 |
| Posterior STS | 23.5 | -12 | 4.5 | 54 |
|  | 18 | -13.5 | -0.5 | 25 |
|  | 26.5 | -14 | 3 | 18 |
| Amy (ABmc) | 6 | -1.5 | -8 | 52 |
| Hippocampus | 7.5 | -4 | -8.5 | 31 |
| PCC (23b) | 3.5 | -12 | 12.5 | 40 |
| Parietal cortex (7b) | 20 | -13.5 | 13.5 | 23 |
| Parietal cortex (PEa) | 11 | -19.5 | 21.5 | 52 |
| Parietal cortex (PEc) | 5 | -25.5 | 22 | 207 |
| IPS (LIP) | 10.5 | -25.5 | 21.5 | 16 |
| Occipital Cortex (V2/V3) | 5 | -32.5 | 15.5 | 177 |
|  | 3.5 | -28 | 18.5 | 17 |
| Occipital Cortex (V4) | 14 | -27.5 | 19.5 | 146 |
| Occipital Cortex (V3) | 11 | -26.5 | 12 | 53 |
|  | 10.5 | -28.5 | 15.5 | 24 |
| Occipital Cortex (V2/V3) | 9.5 | -32.5 | 16 | 64 |
| Occipital Cortex (V2) | 23.5 | -32 | -0.5 | 28 |
|  | 9.5 | -37 | 11 | 115 |
|  | 8.5 | -33 | 19.5 | 71 |
| Cerebellum | 1.5 | -23.5 | 0 | 16 |
|  | 19 | -28.5 | -7 | 50 |
