# Supplementary material for: Behavioral flexibility is associated with changes in structure and function distributed across a frontal cortical network in macaques
Source: PLoS Biol. 2020 May 26;18(5):e3000605. doi: 10.1371/journal.pbio.3000605 (PMC7274449; doi:10.1371/journal.pbio.3000605)
Supplement: S4 Table — Dis Control, discrimination control; DisRev, discrimination reversal; NoDis Control, no-discrimination control. (DOCX) [file pbio.3000605.s007.docx]

**S4 Table : DBM results table: Dis Controls > NoDis Controls (experiment 1, supplementary contrast) Scan 2 > Scan 1**

| Region | x | y | z | Cluster extent (num vox) p < 0.001 |
| --- | --- | --- | --- | --- |
| Ventral lPFC | 19 | 14.5 | 8.5 | 150 |
| LPFC, ventral bank of the principal sulcus | 14.5 | 12 | 8.5 | 413 |
| ACC | 6.5 | 11.5 | 11 | 117 |
|  | 1.5 | 0.5 | 15 | 79 |
| lOFC/cOFC | 13.5 | 12.5 | 4.5 | 19 |
| area 8d/v | 16 | 9.5 | 14 | 294 |
|  | 23 | 7.5 | 5 | 116 |
| F2 6dr/6dc | 20 | 5 | 12 | 130 |
| insula | 19.5 | 6 | 2 | 114 |
| dorsal striatum/fornix | 2.5 | 5 | 6 | 41 |
| IT | 22 | -2 | -5.5 | 730 |
| F2/6Dr/6DC | 12 | 0 | 21 | 50 |
| vm hypothalamus | 2 | -0.5 | -4 | 64 |
|  | 5 | -1 | -4.5 | 50 |
| STS | 18 | -7 | -10 | 333 |
|  | 19.5 | -2.5 | -11 | 72 |
| 3a/b (cs) | 19 | -8 | 11 | 17 |
| 3a/b (cs) ventral gyrus | 22 | -9 | 15 | 158 |
| 3a/b (cs) dorsal gyrus | 16.5 | -7 | 16 | 104 |
| Somatosensory Cortex | 16 | -10.5 | 18.5 | 55 |
| caudal TPO | 20 | -16.5 | 9.5 | 259 |
| SN | 3 | -12 | -9 | 488 |
| PAG | 2.5 | -14.5 | 1 | 63 |
| V4v | 27 | -21.5 | -2.5 | 19 |
| V3d | 7 | -33.5 | 13 | 68 |
|  | 9.5 | -29.5 | 13.5 | 45 |
| V3v | 12 | -16.5 | -8 | 39 |
|  | 20.5 | -27 | 0.5 | 61 |
|  | 11 | -20 | -3.5 | 32 |
|  | 17.5 | -31 | -4.5 | 30 |
| V2/V4v boundary lateral | 23 | -23.5 | 0.5 | 21 |
| V2 medial | 16 | -24.5 | 3.5 | 23 |
| V2 lateral | 22.5 | -24.5 | 6 | 26 |
|  | 17.5 | -29.5 | 15 | 22 |
|  | 19.5 | -27 | 12 | 101 |
| V2 | 8.5 | -29 | 0.5 | 28 |
| V1 | 23.5 | -29 | 5 | 20 |
| V1 | 11.5 | -30.5 | 6 | 16 |
| V1 lateral | 20 | -34 | 6.5 | 48 |
|  | 14 | -36 | -3.5 | 137 |
|  | 17 | -37.5 | 6.5 | 19 |
|  | 18 | -38 | -4 | 19 |
| V1 dorsal | 7 | -36 | 16.5 | 27 |
| Cerebellum | 1.5 | -28.5 | -8.5 | 62 |
|  | 7.5 | -23.5 | -6.5 | 57 |
|  | 16 | -31.5 | -11.5 | 35 |
|  | 9 | -28 | -13 | 88 |
