# Supplementary material for: Behavioral flexibility is associated with changes in structure and function distributed across a frontal cortical network in macaques
Source: PLoS Biol. 2020 May 26;18(5):e3000605. doi: 10.1371/journal.pbio.3000605 (PMC7274449; doi:10.1371/journal.pbio.3000605)
Supplement: S5 Table — OFC, orbitofrontal cortex. (DOCX) [file pbio.3000605.s008.docx]

**Structural changes associated with OFC lesions**

**S5 Table : DBM results table: Lesion study (experiment 2)**

| Region | x | y | z | Cluster extent (num vox) p < 0.001 |
| --- | --- | --- | --- | --- |
| lPFC (12r/46v) | 10.5 | 24.5 | 6.5 | 17 |
| lOFC (12o) | 14.5 | 15 | -2 | 16 |
| MPFC (8Bm) | 2 | 14 | 17.5 | 42 |
| cOFC (11/13) | 7 | 15 | 2 | 6673 |
| Somatosensory cortex (SII) | 22.5 | -2 | 2 | 43 |
| Inferotemporal (TEa) | 14 | 3.5 | -15 | 84 |
| Inferotemporal cortex (TEm) | 26 | -12.5 | -1.5 | 32 |
| mid-STS (PGa) | 17.5 | -1 | -8 | 22 |
| STG | 24.5 | -5 | -5 | 99 |
| Posterior STS | 12.5 | -24.5 | 17.5 | 76 |
| Amygdala (Lv) | 10.5 | -1 | -12.5 | 42 |
| Hippocampus | 15 | -10.5 | -6.5 | 49 |
| PCC (23) | 4 | -13.5 | 9 | 42 |
| Parietal cortex (7a) | 17 | -26 | 16.5 | 21 |
|  | 21 | -18.5 | 14 | 27 |
| Intraparietal sulcus (VIP) | 8.5 | -18.5 | 12 | 74 |
