# Supplementary material for: Behavioral flexibility is associated with changes in structure and function distributed across a frontal cortical network in macaques
Source: PLoS Biol. 2020 May 26;18(5):e3000605. doi: 10.1371/journal.pbio.3000605 (PMC7274449; doi:10.1371/journal.pbio.3000605)
Supplement: S6 Table — OFC, orbitofrontal cortex. (DOCX) [file pbio.3000605.s009.docx]

**Functional changes associated with OFC lesions and discrimination reversal learning (experiment 3 and 4)**

**S6 Table : fMRI-measured activity coupling bilateral Results table:**

| Contrast | Region | x | y | z | Cluster extent (num vox) p < 0.001 |
| --- | --- | --- | --- | --- | --- |
| DisRev Learners > All Controls (experiment 2) Scan 2 > Scan 1 | lOFC (12o) | -12 | 14 | 3 | 17 |
| DisRev Learners > Dis Controls (experiment 2) Scan 2 > Scan 1 | lOFC (12o) | -12 | 14 | 3.5 | 31 |
| OFC Lesion animals < All Controls | lOFC (12o) | 12.5 | 10.5 | -5 | 36 |
|  | Rostral ACC (right) | 2 | 20.5 | 7.5 | 184 |
|  | Rostral ACC (left) | 0 | 20 | 7.5 | 298 |
|  | ACC/MCC (left, 24) | -1.5 | 12.5 | 9.5 | 20 |
